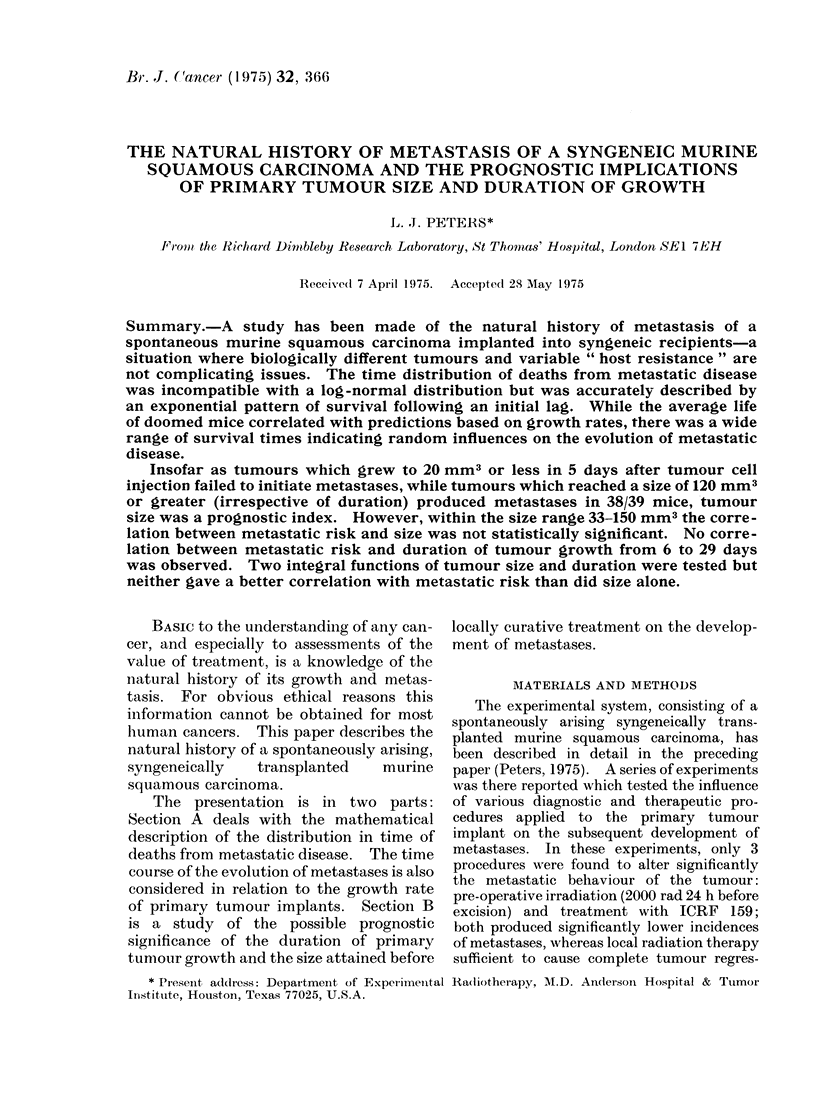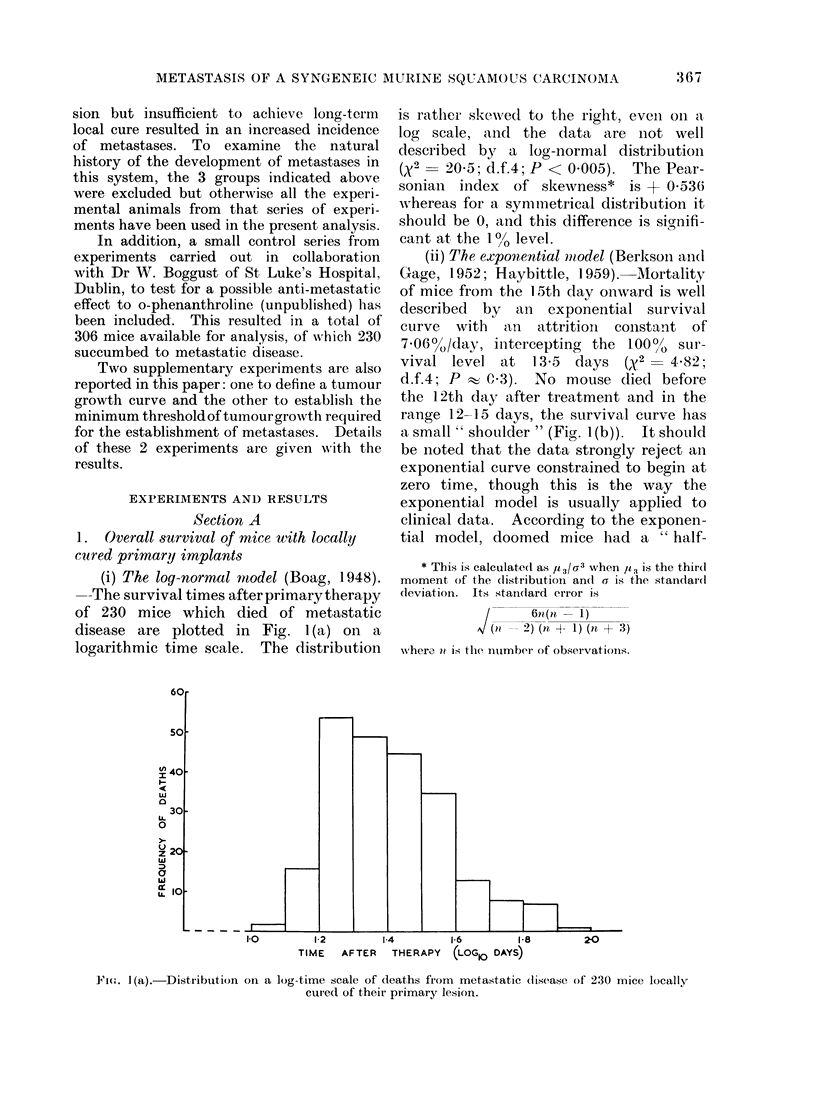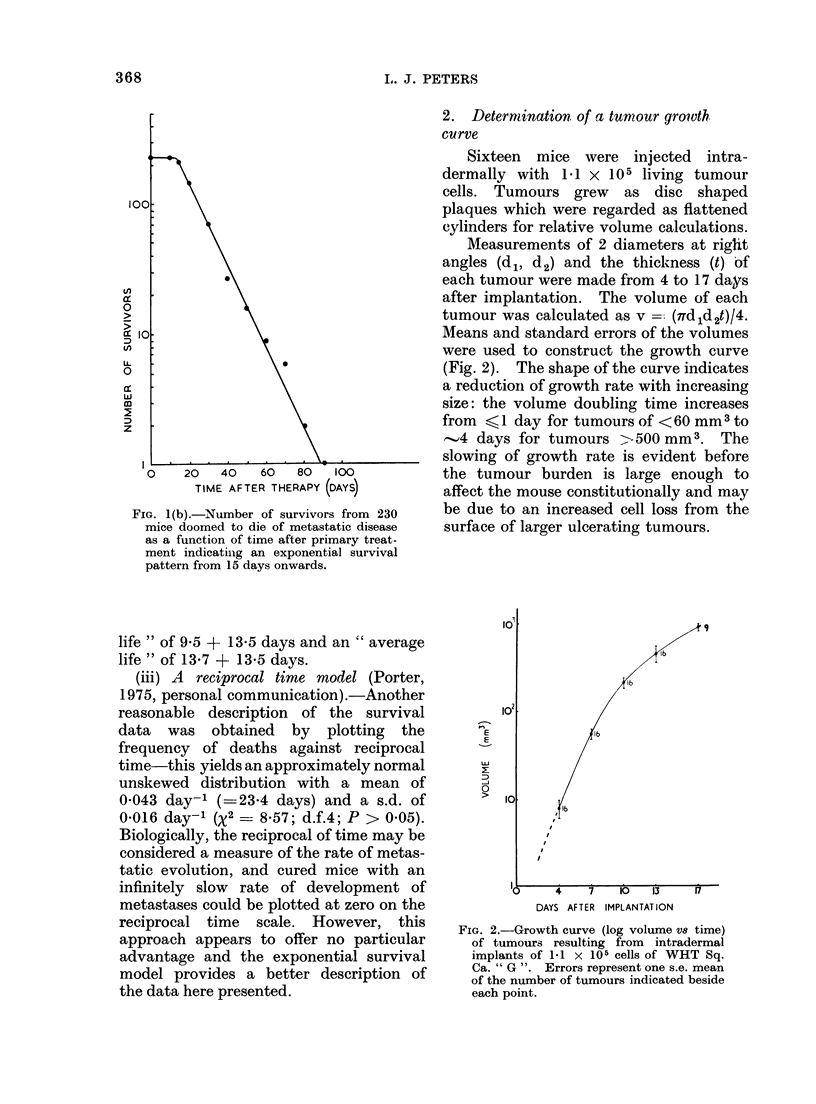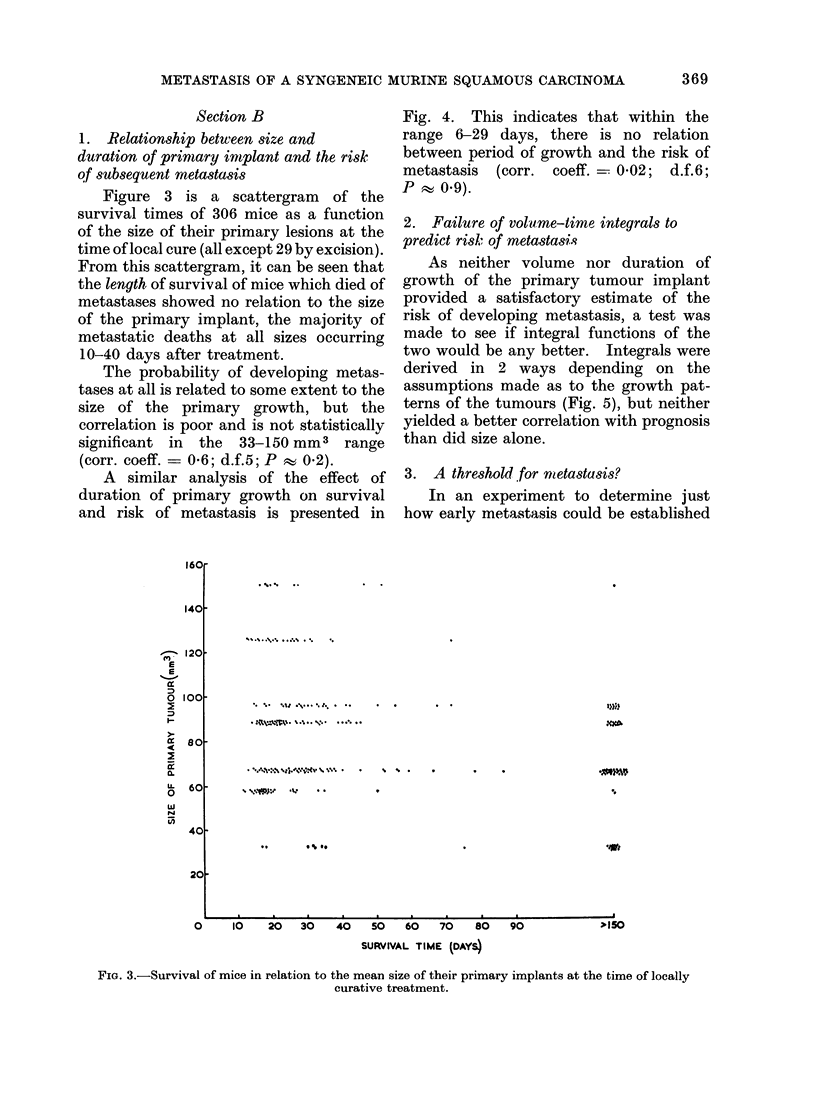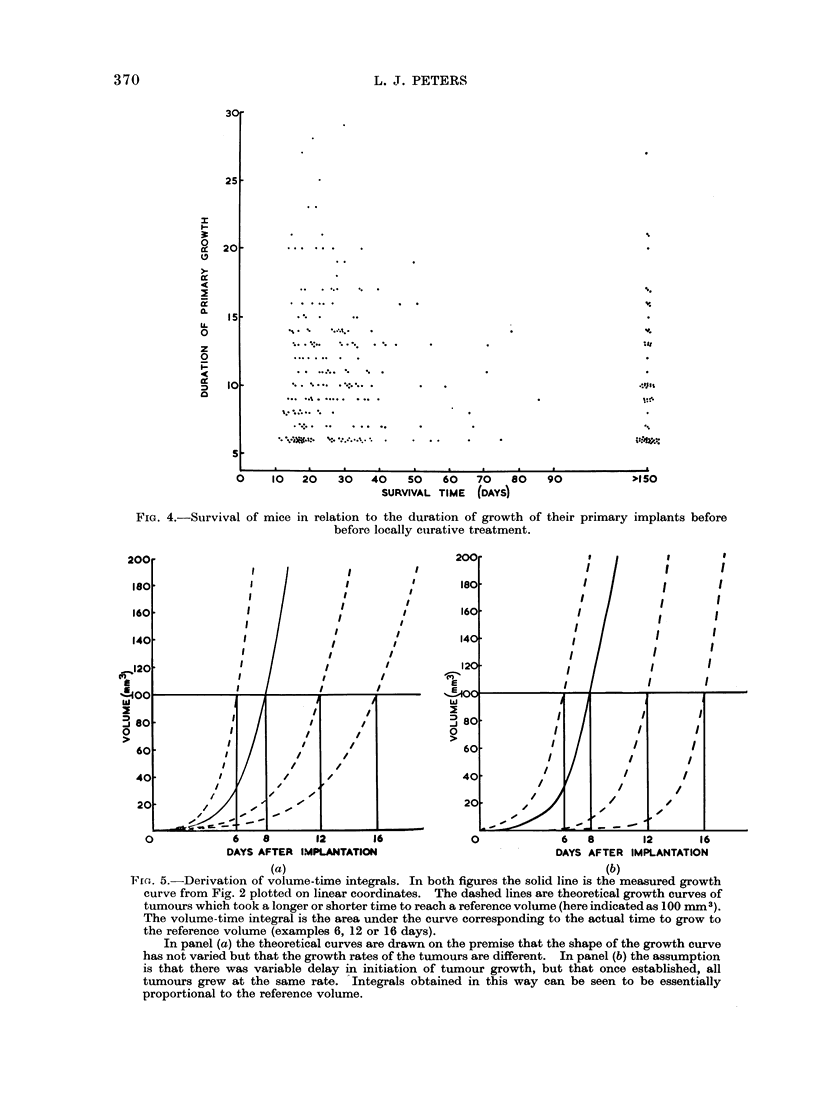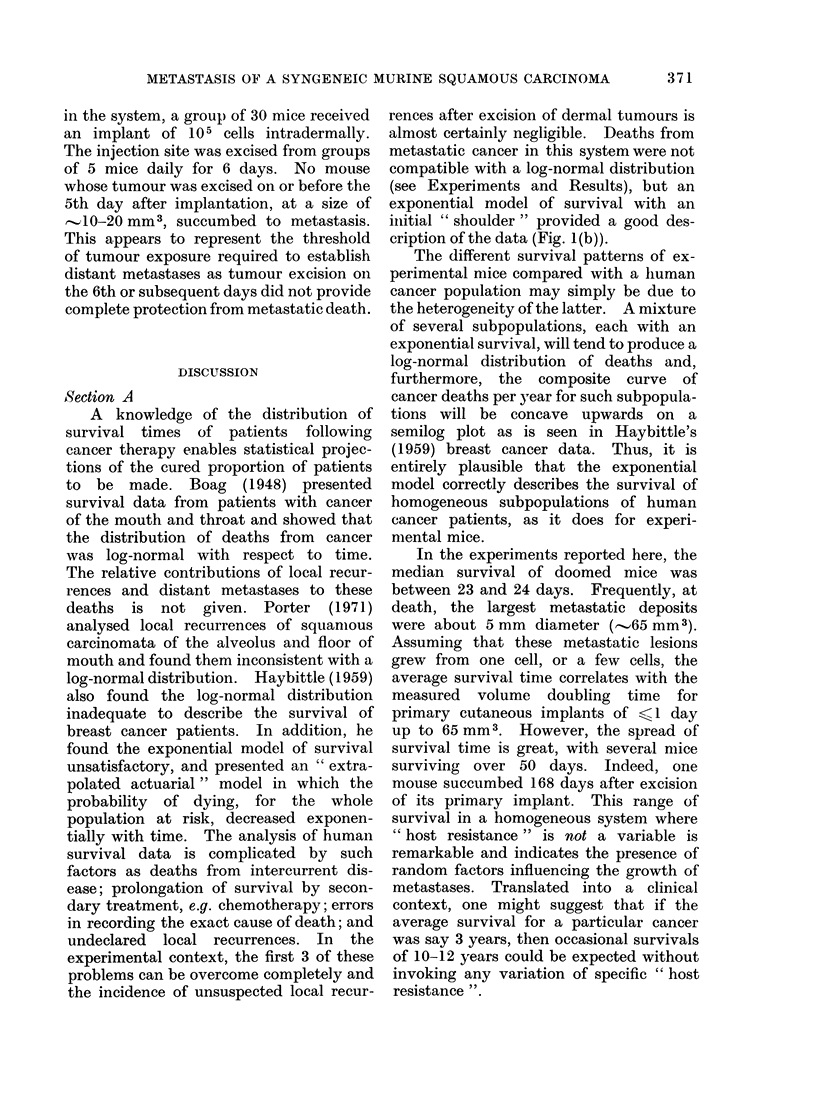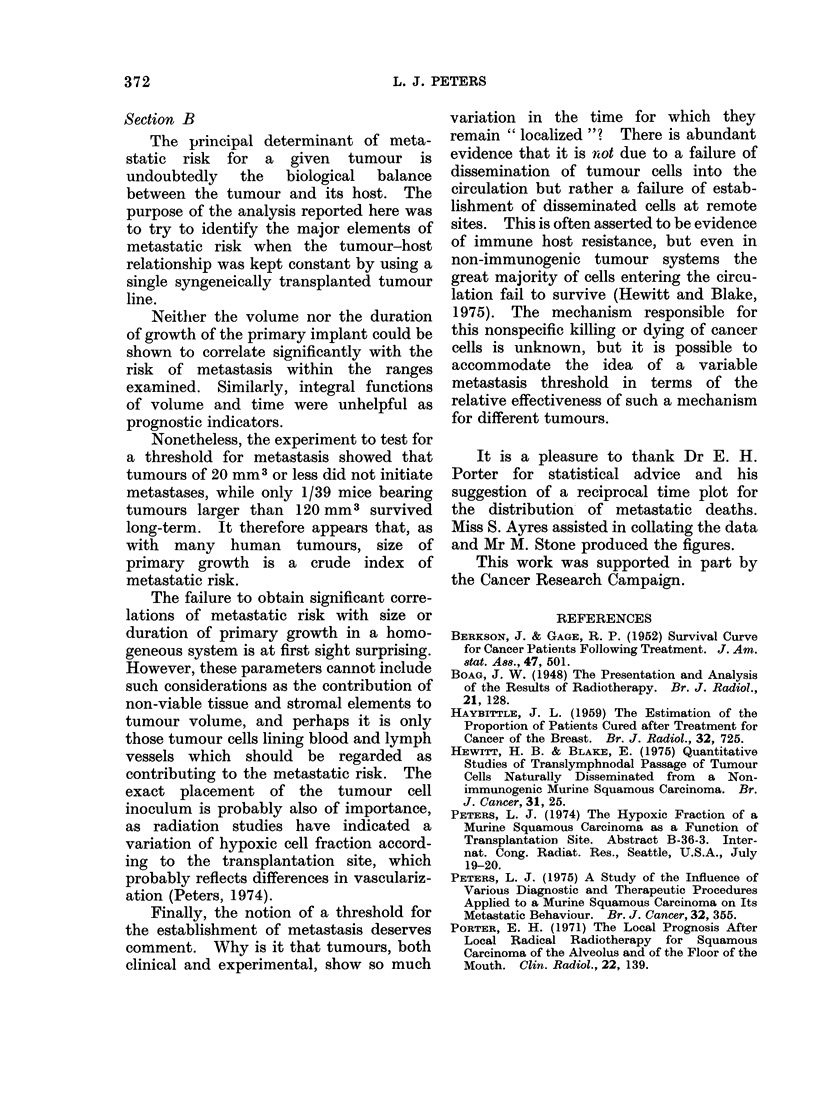# The natural history of metastasis of syngeneic murine squamous carcinoma and the prognostic implications of primary tumour size and duration of growth.

**DOI:** 10.1038/bjc.1975.236

**Published:** 1975-09

**Authors:** L. J. Peters

## Abstract

A study has been made of the natural history of metastasis of a spontaneous murine squamous carcinoma implanted into syngeneic recipients--a situation where biologically different tumours and variable "host resistance" are not complicating issues. The time distrubution of deaths from metastatic disease was incompatible with a log-normal distribution but was accurately described by an exponential pattern of survival following an initial lag. While the average life of doomed mice correlated with predictions based on growth rates, there was a wide range of survival times indicating random influences on the evolution of metastitic disease. Insofar as tumours which grew to 20 mm3 or less in 5 days after tumour cell injection failed to initiate metastases, while tumours which reached a size of 120 mm3 or greater (irrespective of duration) produced metastases in 38/39 mice, tumour size was a prognostic index. However, within the size range 33-150 mm3 the correlation between metastatic risk and size was not statistically significant. No correlation between metastatic risk and duration of tumour growth from 6 to 29 days was observed. Two integral functions of tumour size and duration were tested but neither gave a better correlation with metastatic risk than did size alone.


*Br. J. Cancer* (1975) **32**, 366

### THE NATURAL HISTORY OF METASTASIS OF A SYNGENEIC MURINE SQUAMOUS CARCINOMA AND THE PROGNOSTIC IMPLICATIONS OF PRIMARY TUMOUR SIZE AND DURATION OF GROWTH

L. J. PETERS*

*From the Richard Dimbleby Research Laboratory, St Thomas' Hospital, London SE1 7EH*

Received 7 April 1975. Accepted 28 May 1975

**Summary.—A study has been made of the natural history of metastasis of a spontaneous murine squamous carcinoma implanted into syngeneic recipients—a situation where biologically different tumours and variable " host resistance " are not complicating issues. The time distribution of deaths from metastatic disease was incompatible with a log-normal distribution but was accurately described by an exponential pattern of survival following an initial lag. While the average life of doomed mice correlated with predictions based on growth rates, there was a wide range of survival times indicating random influences on the evolution of metastatic disease.**

**Insofar as tumours which grew to 20 mm$^3$ or less in 5 days after tumour cell injection failed to initiate metastases, while tumours which reached a size of 120 mm$^3$ or greater (irrespective of duration) produced metastases in 38/39 mice, tumour size was a prognostic index. However, within the size range 33–150 mm$^3$ the correlation between metastatic risk and size was not statistically significant. No correlation between metastatic risk and duration of tumour growth from 6 to 29 days was observed. Two integral functions of tumour size and duration were tested but neither gave a better correlation with metastatic risk than did size alone.**

BASIC to the understanding of any cancer, and especially to assessments of the value of treatment, is a knowledge of the natural history of its growth and metastasis. For obvious ethical reasons this information cannot be obtained for most human cancers. This paper describes the natural history of a spontaneously arising, syngeneically transplanted murine squamous carcinoma.

The presentation is in two parts: Section A deals with the mathematical description of the distribution in time of deaths from metastatic disease. The time course of the evolution of metastases is also considered in relation to the growth rate of primary tumour implants. Section B is a study of the possible prognostic significance of the duration of primary tumour growth and the size attained before locally curative treatment on the development of metastases.

### MATERIALS AND METHODS

The experimental system, consisting of a spontaneously arising syngeneically transplanted murine squamous carcinoma, has been described in detail in the preceding paper (Peters, 1975). A series of experiments was there reported which tested the influence of various diagnostic and therapeutic procedures applied to the primary tumour implant on the subsequent development of metastases. In these experiments, only 3 procedures were found to alter significantly the metastatic behaviour of the tumour: pre-operative irradiation (2000 rad 24 h before excision) and treatment with ICRF 159; both produced significantly lower incidences of metastases, whereas local radiation therapy sufficient to cause complete tumour regres-

* Present address: Department of Experimental Radiotherapy, M.D. Anderson Hospital & Tumor Institute, Houston, Texas 77025, U.S.A.

sion but insufficient to achieve long-term local cure resulted in an increased incidence of metastases. To examine the natural history of the development of metastases in this system, the 3 groups indicated above were excluded but otherwise all the experimental animals from that series of experiments have been used in the present analysis.

In addition, a small control series from experiments carried out in collaboration with Dr W. Boggust of St Luke's Hospital, Dublin, to test for a possible anti-metastatic effect to o-phenanthroline (unpublished) has been included. This resulted in a total of 306 mice available for analysis, of which 230 succumbed to metastatic disease.

Two supplementary experiments are also reported in this paper: one to define a tumour growth curve and the other to establish the minimum threshold of tumour growth required for the establishment of metastases. Details of these 2 experiments are given with the results.

### EXPERIMENTS AND RESULTS

### *Section A*

### 1. *Overall survival of mice with locally cured primary implants*

(i) *The log-normal model* (Boag, 1948).—The survival times after primary therapy of 230 mice which died of metastatic disease are plotted in Fig. 1(a) on a logarithmic time scale. The distribution is rather skewed to the right, even on a log scale, and the data are not well described by a log-normal distribution ($\chi^2 = 20{\cdot}5$; d.f.4; $P < 0{\cdot}005$). The Pearsonian index of skewness* is $+\ 0{\cdot}536$ whereas for a symmetrical distribution it should be 0, and this difference is significant at the 1% level.

(ii) *The exponential model* (Berkson and Gage, 1952; Haybittle, 1959).—Mortality of mice from the 15th day onward is well described by an exponential survival curve with an attrition constant of 7·06%/day, intercepting the 100% survival level at 13·5 days ($\chi^2 = 4{\cdot}82$; d.f.4; $P \approx 0{\cdot}3$). No mouse died before the 12th day after treatment and in the range 12–15 days, the survival curve has a small " shoulder " (Fig. 1(b)). It should be noted that the data strongly reject an exponential curve constrained to begin at zero time, though this is the way the exponential model is usually applied to clinical data. According to the exponential model, doomed mice had a " half-

* This is calculated as $\mu_3/\sigma^3$ when $\mu_3$ is the third moment of the distribution and $\sigma$ is the standard deviation. Its standard error is

$$\sqrt{\frac{6n(n-1)}{(n-2)(n+1)(n+3)}}$$

where $n$ is the number of observations.

FIG. 1(a).—Distribution on a log-time scale of deaths from metastatic disease of 230 mice locally cured of their primary lesion.

FIG. 1(b).—Number of survivors from 230 mice doomed to die of metastatic disease as a function of time after primary treatment indicating an exponential survival pattern from 15 days onwards.

life " of 9·5 + 13·5 days and an " average life " of 13·7 + 13·5 days.

(iii) *A reciprocal time model* (Porter, 1975, personal communication).—Another reasonable description of the survival data was obtained by plotting the frequency of deaths against reciprocal time—this yields an approximately normal unskewed distribution with a mean of 0·043 $\text{day}^{-1}$ (=23·4 days) and a s.d. of 0·016 $\text{day}^{-1}$ ($\chi^2$ = 8·57; d.f.4; $P > 0{\cdot}05$). Biologically, the reciprocal of time may be considered a measure of the rate of metastatic evolution, and cured mice with an infinitely slow rate of development of metastases could be plotted at zero on the reciprocal time scale. However, this approach appears to offer no particular advantage and the exponential survival model provides a better description of the data here presented.

2. *Determination of a tumour growth curve*

Sixteen mice were injected intradermally with 1·1 × $10^5$ living tumour cells. Tumours grew as disc shaped plaques which were regarded as flattened cylinders for relative volume calculations.

Measurements of 2 diameters at right angles ($d_1$, $d_2$) and the thickness ($t$) of each tumour were made from 4 to 17 days after implantation. The volume of each tumour was calculated as $v = (\pi d_1 d_2 t)/4$. Means and standard errors of the volumes were used to construct the growth curve (Fig. 2). The shape of the curve indicates a reduction of growth rate with increasing size: the volume doubling time increases from $\leqslant$1 day for tumours of <60 $mm^3$ to ~4 days for tumours >500 $mm^3$. The slowing of growth rate is evident before the tumour burden is large enough to affect the mouse constitutionally and may be due to an increased cell loss from the surface of larger ulcerating tumours.

FIG. 2.—Growth curve (log volume *vs* time) of tumours resulting from intradermal implants of 1·1 × $10^5$ cells of WHT Sq. Ca. " G ". Errors represent one s.e. mean of the number of tumours indicated beside each point.

*Section B*

1. *Relationship between size and duration of primary implant and the risk of subsequent metastasis*

Figure 3 is a scattergram of the survival times of 306 mice as a function of the size of their primary lesions at the time of local cure (all except 29 by excision). From this scattergram, it can be seen that the *length* of survival of mice which died of metastases showed no relation to the size of the primary implant, the majority of metastatic deaths at all sizes occurring 10–40 days after treatment.

The probability of developing metastases at all is related to some extent to the size of the primary growth, but the correlation is poor and is not statistically significant in the 33–150 $mm^3$ range (corr. coeff. = 0·6; d.f.5; $P \approx 0{\cdot}2$).

A similar analysis of the effect of duration of primary growth on survival and risk of metastasis is presented in Fig. 4. This indicates that within the range 6–29 days, there is no relation between period of growth and the risk of metastasis (corr. coeff. = 0·02; d.f.6; $P \approx 0{\cdot}9$).

2. *Failure of volume–time integrals to predict risk of metastasis*

As neither volume nor duration of growth of the primary tumour implant provided a satisfactory estimate of the risk of developing metastasis, a test was made to see if integral functions of the two would be any better. Integrals were derived in 2 ways depending on the assumptions made as to the growth patterns of the tumours (Fig. 5), but neither yielded a better correlation with prognosis than did size alone.

3. *A threshold for metastasis?*

In an experiment to determine just how early metastasis could be established

FIG. 3.—Survival of mice in relation to the mean size of their primary implants at the time of locally curative treatment.

FIG. 4.—Survival of mice in relation to the duration of growth of their primary implants before before locally curative treatment.

FIG. 5.—Derivation of volume-time integrals. In both figures the solid line is the measured growth curve from Fig. 2 plotted on linear coordinates. The dashed lines are theoretical growth curves of tumours which took a longer or shorter time to reach a reference volume (here indicated as 100 $mm^3$). The volume-time integral is the area under the curve corresponding to the actual time to grow to the reference volume (examples 6, 12 or 16 days).

In panel (*a*) the theoretical curves are drawn on the premise that the shape of the growth curve has not varied but that the growth rates of the tumours are different. In panel (*b*) the assumption is that there was variable delay in initiation of tumour growth, but that once established, all tumours grew at the same rate. Integrals obtained in this way can be seen to be essentially proportional to the reference volume.

in the system, a group of 30 mice received an implant of $10^5$ cells intradermally. The injection site was excised from groups of 5 mice daily for 6 days. No mouse whose tumour was excised on or before the 5th day after implantation, at a size of $\sim$10–20 mm$^3$, succumbed to metastasis. This appears to represent the threshold of tumour exposure required to establish distant metastases as tumour excision on the 6th or subsequent days did not provide complete protection from metastatic death.

### DISCUSSION

### *Section A*

A knowledge of the distribution of survival times of patients following cancer therapy enables statistical projections of the cured proportion of patients to be made. Boag (1948) presented survival data from patients with cancer of the mouth and throat and showed that the distribution of deaths from cancer was log-normal with respect to time. The relative contributions of local recurrences and distant metastases to these deaths is not given. Porter (1971) analysed local recurrences of squamous carcinomata of the alveolus and floor of mouth and found them inconsistent with a log-normal distribution. Haybittle (1959) also found the log-normal distribution inadequate to describe the survival of breast cancer patients. In addition, he found the exponential model of survival unsatisfactory, and presented an " extrapolated actuarial " model in which the probability of dying, for the whole population at risk, decreased exponentially with time. The analysis of human survival data is complicated by such factors as deaths from intercurrent disease; prolongation of survival by secondary treatment, *e.g.* chemotherapy; errors in recording the exact cause of death; and undeclared local recurrences. In the experimental context, the first 3 of these problems can be overcome completely and the incidence of unsuspected local recurrences after excision of dermal tumours is almost certainly negligible. Deaths from metastatic cancer in this system were not compatible with a log-normal distribution (see Experiments and Results), but an exponential model of survival with an initial " shoulder " provided a good description of the data (Fig. 1(b)).

The different survival patterns of experimental mice compared with a human cancer population may simply be due to the heterogeneity of the latter. A mixture of several subpopulations, each with an exponential survival, will tend to produce a log-normal distribution of deaths and, furthermore, the composite curve of cancer deaths per year for such subpopulations will be concave upwards on a semilog plot as is seen in Haybittle's (1959) breast cancer data. Thus, it is entirely plausible that the exponential model correctly describes the survival of homogeneous subpopulations of human cancer patients, as it does for experimental mice.

In the experiments reported here, the median survival of doomed mice was between 23 and 24 days. Frequently, at death, the largest metastatic deposits were about 5 mm diameter ($\sim$65 mm$^3$). Assuming that these metastatic lesions grew from one cell, or a few cells, the average survival time correlates with the measured volume doubling time for primary cutaneous implants of $\leqslant$1 day up to 65 mm$^3$. However, the spread of survival time is great, with several mice surviving over 50 days. Indeed, one mouse succumbed 168 days after excision of its primary implant. This range of survival in a homogeneous system where " host resistance " is *not* a variable is remarkable and indicates the presence of random factors influencing the growth of metastases. Translated into a clinical context, one might suggest that if the average survival for a particular cancer was say 3 years, then occasional survivals of 10–12 years could be expected without invoking any variation of specific " host resistance ".

*Section B*

The principal determinant of metastatic risk for a given tumour is undoubtedly the biological balance between the tumour and its host. The purpose of the analysis reported here was to try to identify the major elements of metastatic risk when the tumour–host relationship was kept constant by using a single syngeneically transplanted tumour line.

Neither the volume nor the duration of growth of the primary implant could be shown to correlate significantly with the risk of metastasis within the ranges examined. Similarly, integral functions of volume and time were unhelpful as prognostic indicators.

Nonetheless, the experiment to test for a threshold for metastasis showed that tumours of 20 $mm^3$ or less did not initiate metastases, while only 1/39 mice bearing tumours larger than 120 $mm^3$ survived long-term. It therefore appears that, as with many human tumours, size of primary growth is a crude index of metastatic risk.

The failure to obtain significant correlations of metastatic risk with size or duration of primary growth in a homogeneous system is at first sight surprising. However, these parameters cannot include such considerations as the contribution of non-viable tissue and stromal elements to tumour volume, and perhaps it is only those tumour cells lining blood and lymph vessels which should be regarded as contributing to the metastatic risk. The exact placement of the tumour cell inoculum is probably also of importance, as radiation studies have indicated a variation of hypoxic cell fraction according to the transplantation site, which probably reflects differences in vascularization (Peters, 1974).

Finally, the notion of a threshold for the establishment of metastasis deserves comment. Why is it that tumours, both clinical and experimental, show so much variation in the time for which they remain " localized "? There is abundant evidence that it is *not* due to a failure of dissemination of tumour cells into the circulation but rather a failure of establishment of disseminated cells at remote sites. This is often asserted to be evidence of immune host resistance, but even in non-immunogenic tumour systems the great majority of cells entering the circulation fail to survive (Hewitt and Blake, 1975). The mechanism responsible for this nonspecific killing or dying of cancer cells is unknown, but it is possible to accommodate the idea of a variable metastasis threshold in terms of the relative effectiveness of such a mechanism for different tumours.

It is a pleasure to thank Dr E. H. Porter for statistical advice and his suggestion of a reciprocal time plot for the distribution of metastatic deaths. Miss S. Ayres assisted in collating the data and Mr M. Stone produced the figures.

This work was supported in part by the Cancer Research Campaign.

REFERENCES

Berkson, J. & Gage, R. P. (1952) Survival Curve for Cancer Patients Following Treatment. *J. Am. stat. Ass.*, **47**, 501.

Boag, J. W. (1948) The Presentation and Analysis of the Results of Radiotherapy. *Br. J. Radiol.*, **21**, 128.

Haybittle, J. L. (1959) The Estimation of the Proportion of Patients Cured after Treatment for Cancer of the Breast. *Br. J. Radiol.*, **32**, 725.

Hewitt, H. B. & Blake, E. (1975) Quantitative Studies of Translymphnodal Passage of Tumour Cells Naturally Disseminated from a Non-immunogenic Murine Squamous Carcinoma. *Br. J. Cancer*, **31**, 25.

Peters, L. J. (1974) The Hypoxic Fraction of a Murine Squamous Carcinoma as a Function of Transplantation Site. Abstract B-36-3. Internat. Cong. Radiat. Res., Seattle, U.S.A., July 19–20.

Peters, L. J. (1975) A Study of the Influence of Various Diagnostic and Therapeutic Procedures Applied to a Murine Squamous Carcinoma on Its Metastatic Behaviour. *Br. J. Cancer*, **32**, 355.

Porter, E. H. (1971) The Local Prognosis After Local Radical Radiotherapy for Squamous Carcinoma of the Alveolus and of the Floor of the Mouth. *Clin. Radiol.*, **22**, 139.